# Gestational Diabetes: Which Clinical (Pre)gestational Features Are Able to Predict Failure of Lifestyle Intervention?

**DOI:** 10.7759/cureus.29040

**Published:** 2022-09-11

**Authors:** Patrícia Rosinha, Rosa Dantas, Márcia Alves, Teresa Azevedo, Isabel Inácio, Sara Esteves-Ferreira, Joana Guimarães

**Affiliations:** 1 Endocrinology Department, Centro Hospitalar do Baixo Vouga, Aveiro, PRT

**Keywords:** oral glucose tolerance test, fasting blood glucose, pharmacological therapy, lifestyle intervention, gestational diabetes

## Abstract

Background

Controversy exists regarding risk factors in pregnant women that might be associated with a higher probability of failure of lifestyle intervention in the treatment of gestational diabetes (GD). These pregnant women's risk factors may highlight the need for closer surveillance at an early stage of pregnancy.

Aims

To identify predictors of pharmacological therapy need in early and late GD.

Methods

This was a retrospective observational study including women with GD diagnosed in the first (group 1) or second trimester (group 2) according to the criteria proposed by the International Association of Diabetes Pregnancy Study Group (IADPSG), singleton pregnancy and follow-up between January 2015 and December 2018, divided according to treatment (lifestyle intervention or pharmacological treatment (metformin and/or insulin)).

Results

A total of 278 and 273 women were included in groups 1 and 2, of which 48.6% and 55.3% underwent non-pharmacological treatment, respectively. In group 1, women requiring pharmacological therapy tended to be older and have previous GD or family history of diabetes, higher body mass index (BMI) and higher fasting blood glucose (FBG) levels. In group 2, pharmacological treatment need was associated with multiparity, previous GD, higher BMI, higher fasting glucose value in the oral glucose tolerance test (OGTT), and higher OGTT value at 60 minutes.

The independent risk factors identified for pharmacological treatment requirement were maternal age (OR 1.10 (1.05-1.16), p<0.001), previous GD (OR 2.70 (1.10-6.58), p=0.029) and FBG (OR 1.07 (1.00-1.14), p=0.048) in group 1 while BMI (OR 1.07 (1.02-1.13), p=0.012) and fasting glucose value in the OGTT (OR 1.03 (1.01-1.05), p=0.006) were the factors identified in group 2. The cut-off values for FBG and fasting glucose value in the OGTT that predicted the necessity of pharmacological treatment were 95.50 mg/dL and 88.50 mg/dL, respectively.

Conclusions

In early GD, closer surveillance is necessary for older women with a previous GD and an FBG ≥ 95.50 mg/dL. In late GD, pre-gestational BMI and a fasting glucose value in the OGTT ≥ 88.50 mg/dL should prevail.

## Introduction

Gestational diabetes (GD) is associated with a higher risk of adverse maternal and neonatal outcomes, a fact that inevitably adds importance to its treatment [1]. GD is defined as any degree of hyperglycemia recognized for the first time during pregnancy. Its increased incidence in recent years has been attributed not only to increasing maternal age but also to insulin resistance associated with obesity [2,3].

After decades of first-line use, lifestyle intervention remains a cornerstone in the treatment of GD, although it is not always enough to achieve the recommended glycemic targets. Therefore, in some pregnant women, it will be necessary to consider additional pharmacological treatment (metformin and/or insulin) and the main difficulty is to predict which women will require this approach [4,5].

Previous studies suggest some risk factors possibly associated with greater severity of glucose intolerance, including the presence of a family history of diabetes (first degree), pre-gestational body mass index (BMI), parity, previous GD or macrosomia, maternal age, weight gain at diagnosis or even higher glucose values ​​at diagnosis both in the first or second trimester [5-7]. Moreover, diabetes can be diagnosed at different times of pregnancy, thus being classified into early and late GD, with important implications in terms of outcomes. Pregnant women's risk factors that might be associated with a higher probability of failure to lifestyle intervention in the treatment of GD, highlighting the need for closer surveillance at an early stage of pregnancy, may be different according to the time of diagnosis [1,8,9].

In this way, the present study aims to identify clinical and analytical data associated with the need for pharmacological treatment in early and late GD and predictors of failure of lifestyle intervention. Secondarily, we also intend to compare the obstetric and neonatal outcomes in the lifestyle intervention/pharmacological treatment subgroups.

## Materials and methods

**Figure 1:**
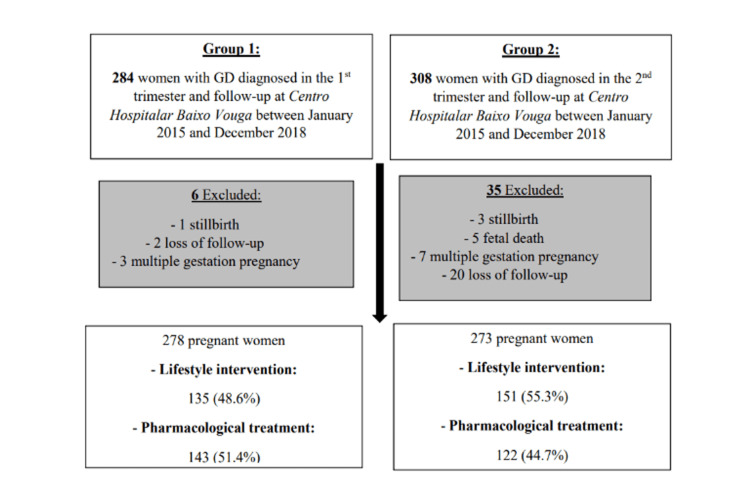
Study flowchart. GD – gestational diabetes.

This was a retrospective observational study including women with a diagnosis of GD and follow-up at Centro Hospitalar Baixo Vouga between January 1, 2015, and December 31, 2018. Exclusion criteria included cases of stillbirth/fetal death, multiple gestation pregnancy, or loss to follow-up (if missing information regarding pregnancy course or obstetric/neonatal outcomes). Figure 1 shows the flowchart of the study. Pregnant women were first divided according to the trimester of GD diagnosis (1st - group 1; 2nd - group 2) and, after applying the exclusion criteria, according to treatment performed during pregnancy (lifestyle intervention/pharmacological treatment). The study protocol was in conformance with the World Medical Association’s Helsinki Declaration and was approved by the Ethics Committee of Centro Hospitalar Baixo Vouga (approval number: 39-02-2021). Informed consent was waived by the ethics committee based on the retrospective nature of the study and full data anonymization.

The following information was collected from the women’s medical records: demographic characteristics, medical and obstetric history, information related to the course of the pregnancy, and perinatal outcomes. Glucose level assessment was performed using the ADVIA® Chemistry Glucose Hexokinase_3 (GLUH_3) assay, with a sensitivity of 4 mg/dL (0.2 mmol/L), adult reference range of 74-106 mg/dL, and coefficient of variation of less than 1%. GD diagnosis was established in the first trimester if fasting blood glucose (FBG) ≥5.1 mmol/L or in the second trimester, using a 75 g oral glucose tolerance test (OGTT) between 24 and 28 weeks of gestation, following the diagnostic criteria proposed by the International Association of Diabetes Pregnancy Study Group (IADPSG): FBG ≥5.1 mmol/L and/or one-hour glucose value ≥10.0 mmol/L and/or two-hour glucose value ≥8.5 mmol/L [10,11]. Lifestyle intervention included: 1) a nutrition consultation to develop an individualized meal plan according to the following macronutrient distribution: 50-55% carbohydrates, 30% fat, and 15-20% protein; 2) recommendations on physical activity: at least 30 minutes of walking daily, preferably in the postprandial period. Pharmacological treatment was considered if it included glucose-lowering therapy, both oral and injectable, in addition to lifestyle intervention.

The following obstetric and neonatal outcomes were analyzed: pre-eclampsia, hydramnios, gestational hypertension, induced labor, cesarean delivery, prematurity (<37 weeks of gestation), macrosomia (birthweight ≥ 4000g), large-for-gestational-age (LGA) infants (defined as birth weight above the 90^th^ percentile of mean weight corrected for fetal sex and gestational age), Apgar score <7, neonatal morbidity (including hypoglycemia, hyperbilirubinemia, respiratory distress syndrome, and/or intensive care unit (ICU) admission), and congenital anomalies.

Data analysis was performed using the statistical package IBM SPSS Statistics, version 20.0 (IBM Corp., Armonk, NY). Categorical variables were presented as frequencies and percentages and continuous variables as mean and standard deviation (SD) or medians and interquartile ranges (IQR) for variables with skewed distribution. Normal distribution was checked using the Shapiro-Wilk test or skewness and kurtosis. Categorical variables were compared with Pearson’s chi-square test or Fisher’s exact test. Continuous variables were compared with the t-test for independent samples or the Mann-Whitney U-test (if skewed distribution). Binary logistic regression was used to evaluate independent factors associated with the need for pharmacological treatment and the receiver operating characteristic (ROC) curve to determine their cut-off values if applicable. Two separate analyses were performed, one for each trimester of GD diagnosis, in which the treatment performed during pregnancy (lifestyle intervention/pharmacological therapy) was considered the dependent variable. Independent variables included in early GD analysis were: maternal age, familiar history of diabetes (1^st^ degree), previous GD, pre-gestational BMI, and FBG. Independent variables included in late GD analysis were: maternal age, previous GD, multiparity, pre-gestational BMI, fasting glucose value in the OGTT, and OGTT value at 60 minutes. All reported p-values are two-tailed, with p<0.05 indicating statistical significance.

## Results

A total of 551 pregnant women met the inclusion criteria for this study; 278 and 273 were respectively included in groups 1 and 2. Regarding the division according to GD treatment, 135 pregnant women of group 1 (48.6%) and 151 of group 2 (55.3%) underwent lifestyle intervention and the remaining pharmacological treatment (Figure 1). Regarding the type of pharmacological treatment performed: a total of 61, 65, and 17 pregnant women of group 1 and 68, 41, and 13 pregnant women of group 2 underwent, respectively, treatment with metformin alone, with insulin alone, or with both.

Table 1 shows maternal characteristics, GD particularities, and obstetric/neonatal outcomes of group 1 categorized according to the treatment modality during pregnancy (lifestyle intervention/pharmacological therapy). Women from the pharmacological treatment group were older (33.4 ± 4.5 years vs 30.2 ± 5.8 years, p<0.001) and had higher medium pre-pregnancy BMI (27.7 (9.9) kg/m^2^ vs 25.3 (6.8) kg/m^2^, p=0.001). Of the 40 (14.4%) women that had GD in a previous pregnancy, eight were managed successfully with a lifestyle intervention while 32 required pharmacological treatment (p<0.001). A total of 45.9% of women from the lifestyle intervention group and 59.4% from the pharmacological treatment group had a first-degree familiar history of diabetes (p=0.030). The median of FBG was significantly higher in women receiving pharmacological therapy (96.0 (7.0) mg/dL vs 94.0 (5.0) mg/dL, p<0.001). In terms of obstetric outcomes, 36.3% of women from the lifestyle intervention group and 54.5% of pharmacologically treated women had induced labor (p=0.002) and, respectively, 27.4% and 30.8% had cesarean deliveries (p=0.408). There were no significant differences in terms of neonatal outcomes (Table 1).

**Table 1:** Maternal characteristics, GD particularities, and obstetric/neonatal outcomes of group 1 (GD diagnosis in the first trimester) categorized according to the treatment performed during pregnancy GD - gestational diabetes. ICU - intensive care unit. IQR - interquartile range. LGA - large-for-gestational-age. SD - standard deviation. YA - younger adults. *Pearson's chi-square. ^a^ - independent samples t-test. ^b^ - Fisher's exact test. ^c^ - Mann-Whitney U test.

	Total (n=278)		Lifestyle intervention (n=135)		Pharmacological treatment (n=143)		p-value
Maternal characteristics							
Maternal age (years), mean ± SD	31.8 ± 5.4		30.2 ± 5.8		33.4 ± 4.5		<0.001^a^
Family history of diabetes (1^st^ degree), n (%)	147	(33.8)	62	(45.9)	85	(59.4)	0.030*
GD in a previous pregnancy, n (%)	40	(14.4)	8	(5.9)	32	(22.4)	<0.001*
Macrosomia in a previous pregnancy, n (%)	18	(6.5)	11	(8.1)	7	(4.9)	0.333*
Multiparity, n (%)	160	(57.6)	72	(53.3)	88	(61.5)	0.183*
Pre-gestational body mass index (Kg/m^2^), median (IQR)	26.5 (8.4)		25.3 (6.8)		27.7 (9.9)		0.001^c^
GD particularities							
Fasting blood glucose (mg/dL), median (IQR)	94.0 (5.0)		94.0 (5.0)		96.0 (7.0)		<0.001^c^
Obstetric outcomes							
Pre-eclampsia, n (%)	8	(2.9)	4	(3.0)	4	(2.8)	1.000^b^
Hydramnios, n (%)	5	(1.8)	2	(1.5)	3	(2.1)	1.000^b^
Gestational hypertension, n (%)	15	(5.4)	4	(3.0)	11	(7.7)	0.111*
Induced labor, n (%)	127	(45.7)	49	(36.3)	78	(54.5)	0.003*
Cesarean delivery, n (%)	81	(29.1)	37	(27.4)	44	(30.8)	0.408*
Neonatal outcomes							
Prematurity, n (%)	25	(9.0)	15	(11.1)	10	(7.0)	0.295*
Birthweight (g), median (IQR)	3140.0 (592.0)		3190.0 (600.0)		3090.0 (576.0)		0.171^c^
Macrosomia, n (%)	10	(3.6)	6	(4.4)	4	(2.8)	0.531^b^
LGA infants, n (%)	20	(7.2)	9	(6.7)	11	(7.7)	0.819*
1-minute Apgar <7, n (%)	14	(5.0)	8	(5.9)	6	(4.2)	0.936^b^
5-minute Apgar <7, n (%)	1	(0.4)	1	(0.7)	0		0.410^b^
Neonatal morbidity, n (%)	50	(18.0)	25	(10.5)	25	(17.5)	0.877*
hypoglycemia	8	(2.9)	6	(4.4)	2	(1.4)	0.164^b^
hyperbilirubinemia	37	(13.3)	18	(13.3)	19	(13.3)	1.000*
respiratory distress syndrome	13	(4.7)	6	(4.4)	7	(4.9)	1.000*
ICU admission	32	(11.5)	17	(12.6)	15	(10.5)	0.708*
Congenital anomalies, n (%)	20	(7.2)	12	(8.9)	8	(5.6)	0.355*

Pregnant women in group 2 who required pharmacological treatment were older (33.8 ± 5.6 years vs 32.3 ± 5.8 years, p=0.028) and more often had a history of GD in a previous pregnancy (18.9% vs 8.6%, p=0.013) (Table 2). Multiparity was significantly more common in pharmacologically treated pregnant women (59.8% vs 45.7%, p=0.004). The median pre-gestational BMI (27.4 (7.0) kg/m^2^ vs 25.1 (6.6) kg/m^2^, p<0.001), fasting glucose value in the OGTT (84.0 (17.0) mg/dL vs 79.0 (15.0) mg/dL, p=0.003), and OGTT blood glucose value at 60 minutes (186.5 (38.0) mg/dL vs 181.0 (35.0) mg/dL, p=0.029) were higher in the pharmacological treatment group. In terms of obstetric outcomes, 21.2% of pregnant women with lifestyle intervention and 32.8% with pharmacological therapy had a cesarean delivery (p=0.038). Neonatal hyperbilirubinemia and LGA infants were more prevalent in the pharmacological treatment group (respectively, 22.1% vs 11.9%, p=0.032; 10.6% vs 3.3%, p=0.025) (Table 2).

**Table 2:** Maternal characteristics, GD particularities, and obstetric/neonatal outcomes of group 2 (GD diagnosis in the 2nd trimester) categorized according to the treatment performed during pregnancy GD - gestational diabetes. ICU - intensive care unit. IQR - interquartile range. LGA - large-for-gestational-age. OGTT - oral glucose tolerance test. SD - standard deviation. *Pearson's chi-square. ^a^ - independent samples t-test. ^b^ - Fisher's exact test. ^c ^- Mann-Whitney U test.

	Total (n=273)		Lifestyle intervention (n=151)		Pharmacological treatment (n=122)		p-value
Maternal characteristics							
Maternal age (years), mean ± SD	33.0 ± 5.8		32.3 ± 5.8		33.8 ± 5.6		0.028^a^
Family history of diabetes (1^st^ degree), n (%)	169	(61.9)	89	(58.9)	80	(65.6)	0.262*
GD in a previous pregnancy, n (%)	36	(13.2)	13	(8.6)	23	(18.9)	0.013*
Macrosomia in previous pregnancy, n (%)	10	(3.7)	6	(4.0)	4	(3.3)	1.000^b^
Multiparity, n (%)	142	(52.0)	69	(45.7)	73	(59.8)	0.004^b^
Pre-gestational body mass index (Kg/m^2^), median (IQR)	26.5 (6.3)		25.1 (6.6)		27.4 (7.0)		<0.001^c^
GD particularities							
Blood glucose in OGTT (mg/dL), median (IQR)							
0 minutes	81.0 (16.0)		79.0 (15.0)		84.0 (17.0)		0.003^c^
60 minutes	183.0 (36.0)		181.0 (35.0)		186.5 (38.0)		0.029^c^
120 minutes	154.0 (40.0)		156.0 (36.0)		152.5 (47.0)		0.428^c^
Obstetric outcomes							
Pre-eclampsia, n (%)	10	(3.7)	6	(4.0)	4	(3.3)	1.000^b^
Hydramnios, n (%)	2	(0.7)	2	(1.3)	0		0.504^b^
Gestational hypertension, n (%)	21	(7.7)	10	(6.6)	11	(9.0)	0.565*
Induced labor, n (%)	112	(41.0)	59	(39.1)	53	(43.4)	0.301*
Cesarean delivery, n (%)	72	(26.4)	32	(21.2)	40	(32.8)	0.038*
Neonatal outcomes							
Prematurity, n (%)	20	(7.3)	13	(8.6)	7	(5.7)	0.485*
Birthweight (g), mean ± SD	3180.9 ± 455.2		3151.2 ± 434.5		3217.6 ± 478.8		0.232^a^
Macrosomia, n (%)	13	(4.8)	5	(3.3)	8	(6.6)	0.258*
LGA infants, n (%)	18	(6.6)	5	(3.3)	13	(10.6)	0.025*
1-minute Apgar <7, n (%)	9	(3.3)	5	(3.3)	4	(3.3)	0.518^b^
5-minute Apgar <7, n (%)	1	(0.4)	1	(0.7)	0		0.181^b^
Neonatal morbidity, n (%)	56	(20.5)	26	(17.2)	30	(24.6)	0.175*
Hypoglycemia	11	(4.0)	5	(3.3)	6	(4.9)	0.548^b^
Hyperbilirubinemia	45	(16.5)	18	(11.9)	27	(22.1)	0.032*
Respiratory distress syndrome	11	(4.0)	6	(4.0)	5	(4.1)	1.000^b^
ICU admission	28	(10.2)	17	(11.3)	11	(9.0)	0.557*
Congenital anomalies, n (%)	22	(8.0)	13	(8.6)	9	(7.4)	0.710*

**Figure 2:**
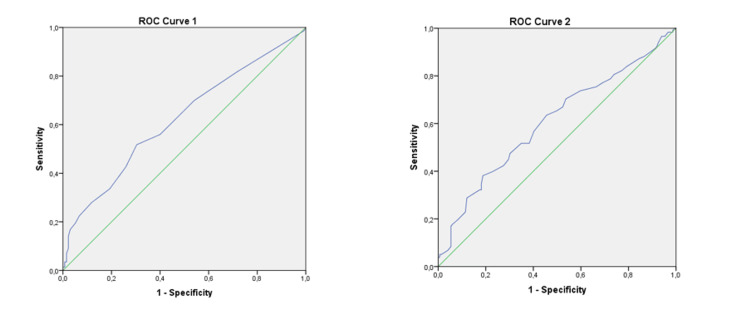
ROC curve 1 showing FPG at the prediction of pharmacological treatment (AUC 0.621; cut-off level 95.5 mg/dL; sensitivity 51.7%; specificity 69.6%; p<0.001). ROC curve 2 showing fasting glucose value in the OGTT at the prediction of pharmacological treatment (AUC 0.607; cut-off level 88.50 mg/dL; sensitivity 38.1%; specificity 81.2%; p=0.003) ROC - receiver operating curve. AUC - area under the curve. FPG - fasting plasma glucose.

The results of univariate and multivariate logistic regression analysis of groups 1 and 2 are presented, respectively, in Table 3 and Table 4. We identified maternal age (OR 1.10 (1.05-1.16), p<0.001), previous GD (OR 2.70 (1.10-6.58), p=0.029) and FBG (OR 1.07 (1.00-1.14), p=0.048) as independent risk factors for the need of pharmacological treatment in early GD (Table 3). The cut-off for FBG to predict the need for pharmacological therapy was 95.50 mg/dL, with a positive predictive value of 62.1% (p<0.001) (Figure 2). Relating to group 2, pre-gestational BMI (OR 1.07 (1.02-1.13), p=0.012) and fasting glucose value in the OGTT (OR 1.03 (1.01-1.05), p=0.006) were identified as independent risk factors for the need of pharmacological therapy in late GD (Table 4). The cut-off point for the fasting glucose value in the OGTT to predict the need for pharmacological treatment was 88.50 mg/dL, with a positive predictive value of 60.7% (p=0.003) (Figure 2).

**Table 3:** Univariate and multivariate logistic regression analysis of independent factors associated with the need for pharmacological therapy in pregnant women of group 1 (GD diagnosis in the 1st trimester) BMI - body mass index. CI - confidence interval. GD - gestational diabetes. OR - odds ratio.

	OR crude	CI 95%	p-value	OR adjusted	CI 95%	p-value
Maternal age	1.128	1.072 - 1.186	<0.001	1.103	1.047 - 1.162	<0.001
Familiar history of diabetes (1st degree)			0.030			0.307
yes	1.726	1.073 - 2.776		1.315	0.789 - 2.224	
no	1			1		
GD in a previous pregnancy			<0.001			0.029
yes	4.577	2.025 - 10.345		2.697	1.105 - 6.585	
no	1			1		
Pre-gestational BMI	1.061	1.018 - 1.107	0.001	1.030	0.986 - 1.076	0.179
Fasting blood glucose	1.101	1.041 - 1.164	<0.001	1.068	1.005 - 1.135	0.035

**Table 4:** Univariate and multivariate logistic regression analysis of independent factors associated with the need for pharmacological treatment in pregnant women of group 2 (GD diagnosis in the 2nd trimester) BMI - body mass index. CI - confidence interval. GD - gestational diabetes. OGTT - oral glucose tolerance test. OR - odds ratio.

	OR crude	CI 95%	p-value	OR adjusted	CI 95%	p-value
Maternal age	1.049	1.005 - 1.095	0.029	1.028	0.979 - 1.079	0.272
GD in a previous pregnancy			0.015			0.101
yes	2.466	1.192 - 5.104		2.010	0.873 - 4.630	
no	1			1		
Multiparity			0.021			0.728
yes	1.770	1.092 - 2.871		1.107	0.625 - 1.961	
no	1			1		
Pre-gestational BMI	1.097	1.043 - 1.153	<0.001	1.071	1.015 - 1.130	0.012
Fasting glucose value in the OGTT	1.033	1.012 - 1.054	0.002	1.031	1.009 - 1.054	0.006
Blood glucose in OGTT at 60 minutes	1.008	1.000 - 1.016	0.061	1.008	0.999 - 1.017	0.081

## Discussion

Our results show that pregnant women with early GD requiring pharmacological treatment clinically differ from those managed successfully with lifestyle intervention; they tend to be older and have a familiar history of diabetes (1^st ^degree), previous GD, higher BMI, and higher FBG levels at diagnosis. Regarding late GD, we found that the need for pharmacological treatment tends to arise in older and multiparous women, with a previous GD, higher pre-gestational BMI values, higher fasting glucose value in the OGTT, and higher OGTT value at 60 minutes. As predictors of lifestyle intervention failure, we found maternal age, previous GD, and FBG for early GD and pre-gestational BMI and fasting glucose values in the OGTT for late GD. The cut-off values for FBG and fasting glucose value in the OGTT to predict the need for pharmacological treatment were, respectively, 95.50 mg/dL and 88.50 mg/dL. In terms of secondary outcomes, we found an association between pharmacological treatment and induced labor in pregnant women with early GD; cesarean delivery, LGA infants, and neonatal hyperbilirubinemia in late GD.

Interestingly, the percentage of pregnant women requiring pharmacological treatment was higher in the group with early GD. Previous studies suggest that a diagnosis of GD in the first trimester may be an indicator of the greater severity of maternal metabolic dysfunction, possibly affecting the fetus over a longer period of time or at an earlier stage of development. Despite this theory, most of the current evidence does not demonstrate significant differences in terms of neonatal outcomes when comparing infants born to mothers with early and late GD [9]. In our study, a pharmacological intervention was more frequent in the group with early GD, allowing rapid metabolic control, which might explain the lack of direct negative impact on neonatal outcomes.

Pre-gestational BMI only had a predictive value of failure to lifestyle intervention in late GD, contrary to expectations. This might be related to the prevalence of overweight in the samples, which was higher in the group diagnosed with late GD (60.8% vs 58.3%). Current evidence gives importance to pre-gestational BMI as an essential factor in the prevention of adverse pregnancy outcomes [11], and its association with a higher probability of pharmacological therapy requirement, independently of gestational weight gain, confirms BMI has a central role in the preconception period as a modifiable factor.

In early GD, according to our results, maternal age, previous GD, and FBG ≥ 95.50 mg/dL may be used as independent risk factors for the need for pharmacological treatment while, in late GD, the pre-gestational features able to predict failure of lifestyle intervention include pre-gestational BMI and, analytically, a fasting glucose value in the OGTT ≥ 88.50 mg/dL. It is also worth noting that, in both early and late GD, the only analytical criterion with a predictable value corresponded to a measurement of fasting glucose, which raises important questions regarding the relevance of the OGTT in the diagnosis and management of this condition.

This study stands out for its relevance both in clinical terms and for economic reasons. Metabolic control in GD is a relevant issue because it translates into an improvement in maternal and fetal outcomes, especially when achieved at an early stage of pregnancy. However, pharmacological treatment of this condition, despite being safe, entails non-negligible costs that must also be considered [6]. Thus, the results of this study provide important clues for the correct selection of women who will need pharmacological treatment, allowing a more cost-effective approach to the disease.

A few limitations should be noted. First is its retrospective nature, which does not allow ensuring correct compliance with the proposed treatment, with a possible bias in the results presented. We also acknowledge that, despite the adequate overall sample size, the subdivision into groups might have decreased statistical power. Moreover, cases of fetal death and stillbirth were not considered. 

## Conclusions

In conclusion, in early GD, maternal age, previous GD, and FBG equal to or greater than 95.50 mg/dL may be used as independent risk factors for the need for pharmacological treatment while in late GD the pre-gestational features able to predict the failure of lifestyle intervention are pre-gestational BMI and a fasting glucose value in the OGTT equal to or greater than 88.50 mg/dL These are, according to our results, the main factors to be considered as clues to decide which pregnant women will need closer surveillance. We hope that these results can be a step forward not only for the improvement of health care provided in GD but also for more effective management of available resources.
